# In vivo pair correlation microscopy reveals dengue virus capsid protein nucleocytoplasmic bidirectional movement in mammalian infected cells

**DOI:** 10.1038/s41598-021-03854-z

**Published:** 2021-12-24

**Authors:** Ignacio Sallaberry, Alexis Luszczak, Natalia Philipp, Guadalupe S. Costa Navarro, Manuela V. Gabriel, Enrico Gratton, Andrea V. Gamarnik, Laura C. Estrada

**Affiliations:** 1 Departamento de Física, Facultad de Ciencias Exactas y Naturales, Universidad de Buenos Aires and IFIBA-National Research Council for Science and Technology (CONICET), 1428 Buenos Aires, Argentina; 2 Fundación Instituto Leloir-National Research Council for Science and Technology (CONICET), 1405 Buenos Aires, Argentina; 3 Laboratory for Fluorescence Dynamics and Beckman Laser Institute and Medical Clinic, University of California, Irvine, CA USA

**Keywords:** Biophysics, Molecular biology

## Abstract

Flaviviruses are major human disease-causing pathogens, including dengue virus (DENV), Zika virus, yellow fever virus and others. DENV infects hundreds of millions of people per year around the world, causing a tremendous social and economic burden. DENV capsid (C) protein plays an essential role during genome encapsidation and viral particle formation. It has been previously shown that DENV C enters the nucleus in infected cells. However, whether DENV C protein exhibits nuclear export remains unclear. By spatially cross-correlating different regions of the cell, we investigated DENV C movement across the nuclear envelope during the infection cycle. We observed that transport takes place in both directions and with similar translocation times (in the ms time scale) suggesting a bidirectional movement of both C protein import and export.

Furthermore, from the pair cross-correlation functions in cytoplasmic or nuclear regions we found two populations of C molecules in each compartment with fast and slow mobilities. While in the cytoplasm the correlation times were in the 2–6 and 40–110 ms range for the fast and slow mobility populations respectively, in the cell nucleus they were 1–10 and 25–140 ms range, respectively. The fast mobility of DENV C in cytoplasmic and nuclear regions agreed with the diffusion coefficients from Brownian motion previously reported from correlation analysis. These studies provide the first evidence of DENV C shuttling from and to the nucleus in infected cells, opening new venues for antiviral interventions.

## Introduction

Dengue virus (DENV) is the most significant arthropod-borne virus infecting humans around the world^1,2^. Despite the urgent need of controlling DENV infections, lack of understanding of the primary functions of key proteins and their interactions with the host cell strongly limits the development of antiviral drugs. The capsid protein (C protein), among other viral proteins, plays crucial roles in multiple processes, including structural maintenance, virus particle assembly, and release of the viral genome during infection^3,4^, and thus has been identified as an attractive candidate for antiviral strategies^5^. C protein of various flaviviruses has been previously shown to enter the nucleus of infected cells; however, whether the transport by which C protein is transported occurs from and to the nucleus is still unknown.

Fluorescence correlation spectroscopies are ideal techniques to study the behavior of single proteins in live, intact cells. The pair Correlation Function (pCF) approach is one of the correlation-based techniques that has grown in the last years to study single molecule mobility. As originally conceived by Digman^6^ and more recently applied to the 2D case^7,8^, the pCF analysis in 1 and 2 dimensions calculates the spatiotemporal cross-correlation of the fluorescence intensity among two temporally and spatially separated pixels extracting the time needed for each molecule to be found in a position different from the one it has at the starting time. If molecules found barriers to diffusion that need to be crossed to reach their final position, a longer time will be needed for the same molecule to be found at a position across the barrier. The pCF approach has proven to be a powerful tool to study molecular shuttling across the NE^9^, mobility within intracellular structures or obstacles such fibers or filaments (chromatin, actin, or microtubules)^10^, microdomains (as the endoplasmic reticulum, ER), organelles, lipid droplets, among others.

Here, we investigated the DENV C protein movement across the nuclear envelope during the infection cycle. Our data supports transport of the viral protein across the nuclear membrane in both directions. The times observed for the movement of C across the membrane were in the ms time scale for both import and export from the nucleus. Additionally, from the pair cross-correlation functions in the cytoplasm or nucleus of infected-cells, we found two populations of C molecules with different mobilities in each compartment.

## Results and discussion

### DENV capsid-protein mobility in infected cells

**Figure 1:**
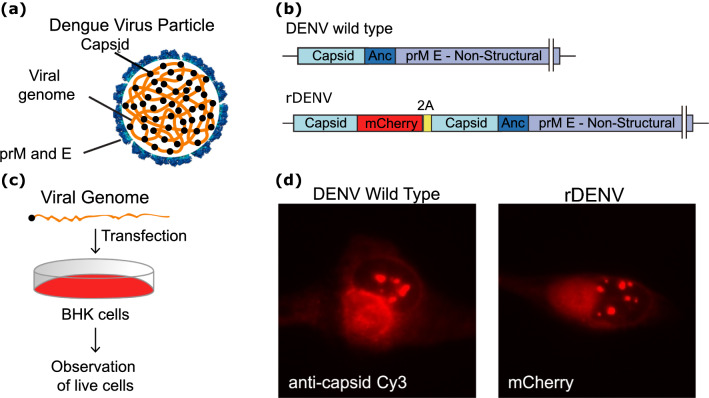
Construction of the recombinant full-length DENV containing the C-mCherry fusion. (**A**) Scheme of a DENV particle showing the viral genome (orange), the capsid protein (black), and the lipid membrane containing the pro-membrane (prM) and envelope (E) proteins. (**B**) Schematic of the DENV virus genome (DENV wild type) and the recombinant DENV (rDENV) carrying the capsid sequence fused to mCherry, followed by the foot and mouth disease virus 2A auto cleavage site. The complete sequence of the viral genome is indicated as prM E—Non-Structural region. (**C**) Schematic of the protocol of viral RNA transfection. (**D**) Immunofluorescence using anti-capsid antibodies (DENV wild type) and C-mCherry visualization (DENV C-mCherry), both 24 h post-RNA transfection.

To quantitatively analyze the mobility of the DENV C protein during the infectious viral cycle, we generated a recombinant full-length viral genome encoding a C protein fused to the mCherry coding sequence, in the context of a duplicated C-coding region to ensure the function of RNA cis-acting elements. Transfections of transcribed viral RNA into mosquito or mammalian cells led to translation and amplification of the viral genome. To examine whether the fusion C-mCherry protein behaved as the WT C protein, we performed viral RNA transfections with the WT and the recombinant viral genomes (rDENV). Immunofluorescence analysis using specific antibodies against C protein for the WT virus reproduced that observed with the C-mCherry confocal images showing C-mCherry accumulation in the cytoplasm, nucleolus, and lipid droplets of the infected cells (Fig. 1 and supplementary information). We conclude that C-mCherry is suitable to investigate C protein nucleocytoplasmic shuttling and intracompartment dynamics during a viral replication cycle.

**Figure 2:**
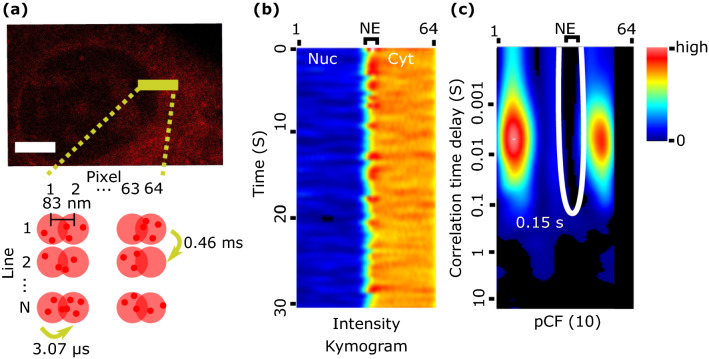
Schematic representation of the pCF analysis. A 64-pixels long line (corresponding to a distance of 5.3 μm) across NE is repeatedly scanned from left-to-right with a line time of 0.46 ms, a pixel dwell time of 3.07 μs, and a pixel size of 83 nm (resulting in an oversampling of the microscope’s PSF, pink circles). (**A**) A confocal image of an infected cell. Scale bar is 5 μm. (**B**) Fluorescence intensity along lines is collected and represented as a kymogram where pixels along the lines correspond to the horizontal axis while the time is in the vertical axis. The position of the NE is identified with a resolution of 200—300 nm (2–6 pixels depending on experimental pixel size) as it is diffraction limited. (**C**) The pair correlation function at a distance of 10 pixels (830 nm) is calculated pixel-by-pixel and represented with the pixel position in the horizontal axis and the time in logarithmic scale in the vertical axis. The white arch indicates a lengthening in the transport throughout the NE.

To study C protein mobility, we used confocal line scanning measurements followed by a pair correlation analysis of pixels along the scanned lines. Each experiment consisted of 200,000 consecutive lines (approx. 100 s) of 64-pixels each, at a line rate of 0.46 ms/line and a pixel size of 83 nm, which resulted in an oversampling of the microscope point spread function (PSF), Fig. 2A. Sequentially plotting the acquired lines generates a kymogram (or carpet) map composed of 200,000 rows and 64-columns, where the horizontal and vertical coordinates represent pixels and time respectively (Fig. 2B). Raster line scanning experiments were performed on BHK cells transiently expressing mCherry from a plasmid (control experiments) or transfected with DENV C-mCherry RNA. In all measured cells, the scanned line was chosen to perpendicularly cross the nuclear envelope (NE) interface and to maintain its position at the center of the line. Thus, the first half of the pixels along the lines corresponds to one compartment (e.g., the cytoplasm) and the second half of pixels corresponds to the other compartment (e.g., the nucleus), as schematically shown in Fig. 2B. Finally, the pair correlation function at a distance of 10 pixels was calculated and represented in a kymogram format with the pixel in the horizontal, and the time in logarithmic scale in the vertical directions (Fig. 2C). In both cases, kymograms are color-coded from black (zero amplitude) to red (highest amplitude).

After pCF analysis, we observed a visible arch (marked in white in the kymogram), coincident with the position of the NE, thus indicating a lengthening in the transport time throughout the NE. Note that since the pCF(δr) correlates the intensity in every pixel, r, with the intensity in pixel r + δr, the pCF(10) kymogram provides information only from column 1 to column 54. The kymograms are color-coded from black (zero amplitude) to red (highest amplitude).

Finally, a careful visual inspection of all data sets was done before the pCF analysis to determine if an intensity correction procedure due to photobleaching, and/or the analysis of only a subset of the entire measured lines were necessary as previously described^6^. This pre-inspection of the measured data is a crucial step to detect photobleaching or other intensity drifts (including cellular movements) that are likely to occur during the time scale of one experiment (approx. 100 s) and which may interfere on the pCF curves calculations and further interpretation.

### Intra-compartment mobility of the DENV C protein

**Figure 3:**
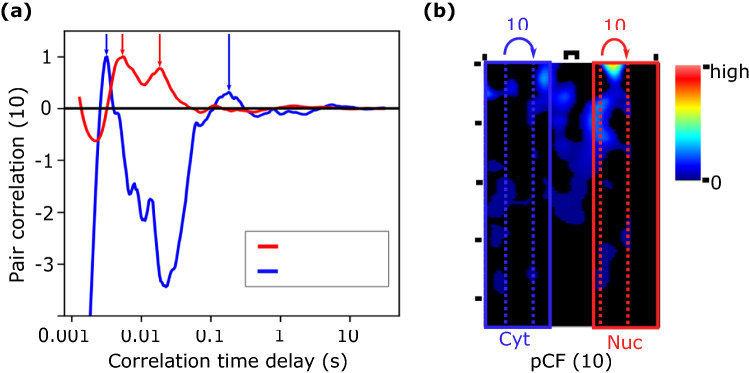
pCF analysis of cytoplasm-to-cytoplasm (blue) and nucleus-to-nucleus (red) C-DENV mobility shows two peaks in the pCF function suggesting the presence of two protein populations with different mobilities. (**A**) Averaged normalized pCF(10) curves show that C protein moves faster in the cytoplasm ($t_{cyto}^{ short} = 3\,{\text{ms}}$, corresponding to an apparent diffusion coefficient of approx. 40 μm^2^/s) than in the nuclear compartment ($t_{ nucleus}^{ short} = 6\,{\text{ms}}$, corresponding to an apparent diffusion coefficient of approx. 20 μm^2^/s). Interestingly, both compartments show two positive peaks in the pCF function (two correlation time delays), suggesting the presence of two protein populations with different mobilities. Peaks in the pCF with negative values were not included in the analysis as they correspond to anti (minimum) or non (maximum) signal correlations. (**B**) pCF(10) kymogram. Warmer colors indicate higher pCF amplitudes. Superimposed a schematic representation of the intra-compartment pCF analysis where cytoplasmic and nuclear zones are indicated by blue and red rectangles respectively.

As a first approach to investigate C protein dynamics in live-infected-cells, we studied the intra-compartment (cytoplasm-to-cytoplasm and nucleus-to-nucleus) protein mobility. The pCF(8) and pCF(10), corresponding to distances of 664 and 830 nm respectively, were computed excluding in all cases the 5 pixels (approx. 400 nm) close to the NE. The region around the NE is expected to be strongly influenced by the nuclear membrane and hence we excluded it from the analysis. Then, to determine the mobility of C protein in cytoplasm and nucleus, we computed the pCF curves and found the times of maximum positive correlation (minima and negative maxima, while interesting, are outside the scope of this work). Figure 3 shows a typical example where the normalized pair cross-correlation functions corresponding to intra-cytoplasmic (blue curve) and intra-nuclear (red curve) were computed at a distance of 10 pixels (830 nm) and at 3 h post RNA-transfection. Interestingly, in both compartments we found that two correlation time delays (two maxima in the pCF curves) appeared, showing the presence of two protein populations with different mobilities. In the example shown in Fig. 3, the fast mobility protein population (corresponding to the short correlation time delay) moves in the cytoplasm with a correlation time $t_{cyto}^{ short} = 3\,{\text{ms}}$, corresponding to an apparent diffusion coefficient of approx. 40 μm^2^/s and with a correlation time $t_{ nucleus}^{ short} = 6\,{\text{ms}}$, corresponding to an apparent diffusion coefficient of about 20 μm^2^/s in the nuclear compartment.

**Figure 4:**
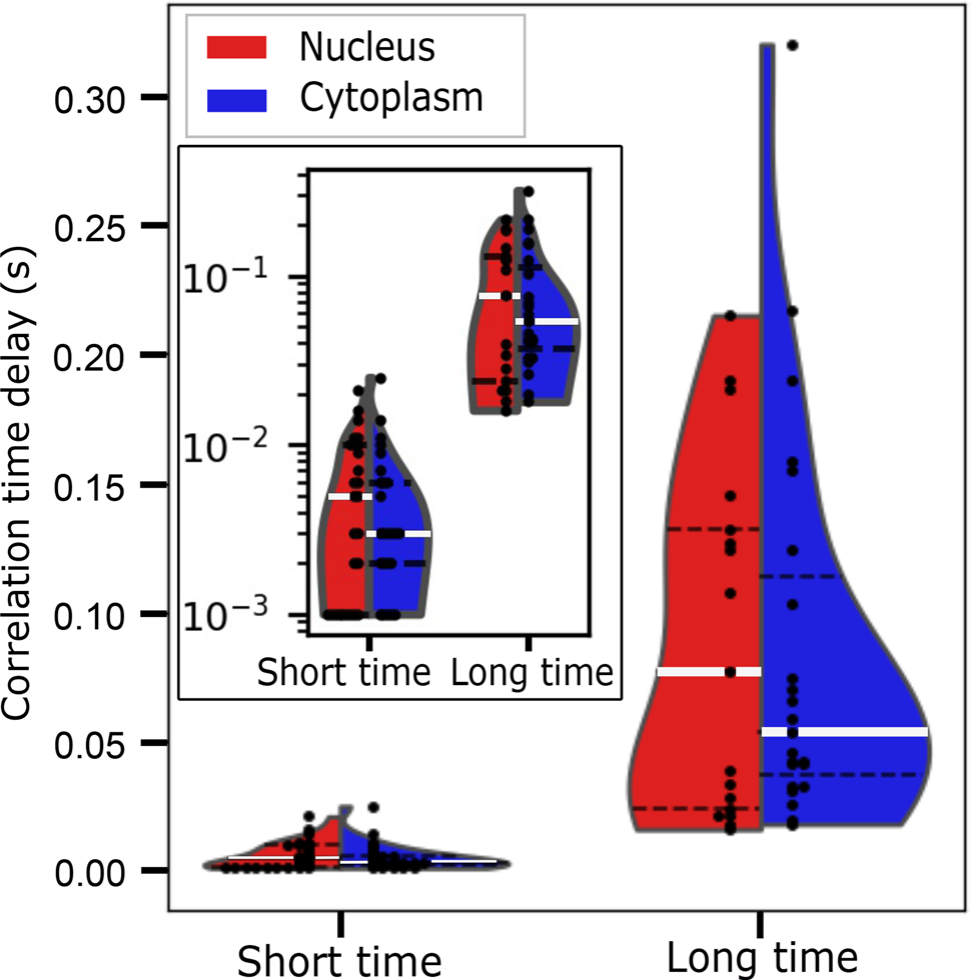
Measured correlation times show 2 different C protein mobility components. Violin plots show the distribution of the correlation times for the fast and slow C protein mobility components in the nucleus (red) and cytoplasm (blue) of DENV infected cells. In both nucleus and cytoplasm compartments, fast and slow mobility components were identified. While the fast mobility component (short correlation times) has a narrow time distribution, the slow mobility group (long correlation times) has a broader distribution of the obtained correlation times delays. The black points indicate measured times, the white solid lines indicate the median and the black dashed lines the 25% and 75% percentiles. Inset: same data shown in logarithmic scale.

To further analyze the viral C protein mobility and investigate the origin of the second correlation time (second peak in the pCF curves), we analyzed 17 measurements from at least 5 independent transfection experiments. In all the analyzed cases, we found two populations of DENV C-protein characterized by both a fast (median time in the ms range) and a slow (median time in the hundreds of ms range) correlation times. Figure 4 shows a violin plot of the correlation times distribution and the probability density of each protein population revealing that for both compartments (cytoplasm in blue and nucleus in red) the distribution of the correlation times for the slow and fast mobility components are substantially different (Mann–Whitney test: U_short cyto-long cyto_ = 2, p_short cyto-long cyto_ = 1.9 10^–9^; U_short nuc-long nuc_ = 4, p_short nuc-long nuc_ = 2.4 10^–8^). While the high mobility component (short correlation times delays) has a narrow distribution of the data, the low mobility group (long correlation times delays) has a broader distribution of the obtained times. Although different factors may influence the behavior of the viral protein intracellular mobility, the two peaks observed in more than 60% of the average pCFs calculated curves (18 from 30 and 23 from 30 in the nuclear and cytoplasmic compartments, respectively) suggest different diffusion dynamics across subcellular regions due to obstacles and/or interactions between C protein and cellular components, as previously suggested for other models^11^.

In all experiments, we explored lines of 5 to 6 μm long crossing the NE. Thus, the studied cytoplasmic region mainly corresponds to the ER, while the studied nuclear region corresponds to the nucleoplasm. According to this scenario, we postulate that the observed short correlation times with medians $t_{ nucleus}^{ short} = 5\,{\text{ms}},$ and $t_{cyto}^{ short} = 3\,{\text{ms}},$ (corresponding to apparent diffusion coefficients of Dapp_nuc = 23 μm^2^/s and Dapp_cyto = 38 μm^2^/s) are compatible with C protein freely diffusing in the nucleus and cytoplasm of infected-cells, while the long correlation times with medians $t_{ nucleus}^{ long} = 77\,{\text{ms}},$ and $t_{ cyto}^{ long} 54\,{\text{ms}},$ (corresponding to apparent diffusion coefficients of Dapp_nuc = 1 μm^2^/s and Dapp_cyto = 2 μm^2^/s) are consistent with C protein probably interacting to subcellular components. As the C protein interactions can occur with different components inside the 3D crowded cell interior, it is reasonable to observe a broader distribution of correlation times for the slow mobile (long correlation times) fraction of proteins as observed in Fig. 4 linear scale.

**Figure 5:**
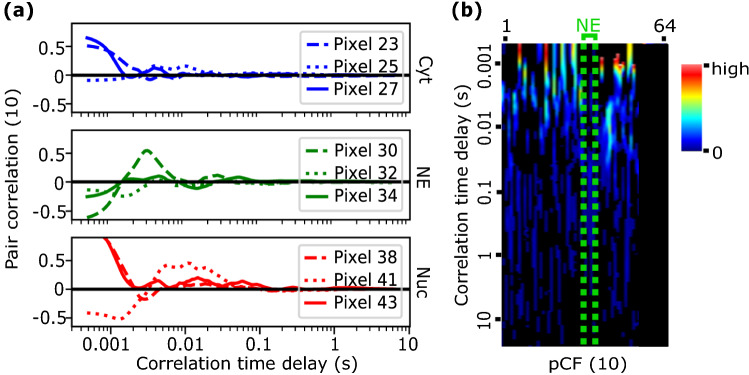
pCF pixel-by-pixel analysis shows intrinsic variability in the cell interior. Representative results of a pCF analysis calculated pixel-by-pixel along a scanned confocal line that crossed the cytoplasm, the nucleus, and the nuclear envelope of a DENV infected cell. In this example the position of the NE is approximately at pixel 33. As expected, a plot of the computed pCF curves show differences among pixels since several intra-compartment obstacles to isotropic diffusion can be detected along different positions (pixels) of the lines. (**A**) pCF(10) curves at specific pixels (indicated in the figure label) are displayed in different colors: cytoplasm-to-cytoplasm (blue), cytoplasm-to-nucleus (green), and nucleus-to-nucleus (red) lines. (**B**) The pixel-by-pixel pCF(10) kymogram shows a variability in the curves at different positions (pixels) of the scanned lines. The minimum value for the color scale (black) was set to zero amplitude of the pCF(10). Thus, all negative pCFs points are represented in black and correspond to no communication.

All obtained pCF curves corresponding to intra-compartment mobility show that the main mechanism of C protein dynamics follows a free diffusion pattern, with measured correlation time delays of a few milliseconds (diffusion coefficients in the 10 to 40 μm^2^/s range). Considering the fast mobility component only, the median correlation times in the cytoplasm is shorter than in the nucleus and, although a Mann–Whitney test shows the difference is not statistically significant (U_short cyto-short nuc_ = 328, p_short cyto-short nuc_ = 0.430), this trend agrees with our previous results using RICS analysis^7^. Moreover, since several intra-compartment obstacles to isotropic diffusion can be detected along different experiments or along different positions (columns) during a set of lines, obtaining similar pCF curves at different positions (pixels) of the line even at the same data set is not expected^12^. This is what we observed for the pCF curves calculated at different positions (the cytoplasm region in blue, the nucleus in red and crossing the NE in green) across the measured lines (Fig. 5).

**Figure 6:**
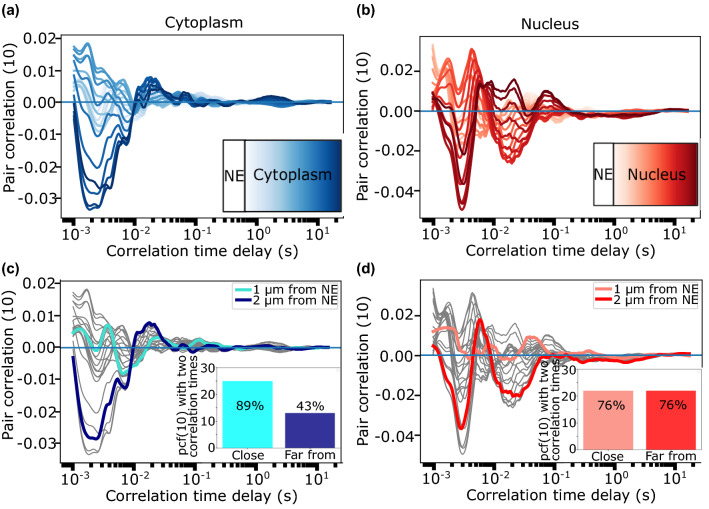
pCF pixel-by-pixel analysis Representative results of a pCF(10) analysis calculated pixel-by-pixel along a scanned confocal line across the cytoplasm (left) and the nucleus (right) of a DENV infected cell. Panels (**A**,**B**) show pCF(10) curves as a function of the NE distance (the closer to the NE, the lighter the color). Pixels closer to each other (similar shades of blue or red) tend to have similar correlation curves. In panels (**C**,**D**) pCF(10) curves at 1 and 2 μm from NE are highlighted showing the presence of positive correlation peaks close to the NE (in light color) but the presence of only one positive correlation peak far from the NE (in dark color).

To further analyze the differences among pCF curves computed at different positions (pixels) of a line experiment, a representative complete data set is shown in Fig. 6. pCF(10) curves that correlate cytoplasm to cytoplasm pixels show gradual shape changes with the pixel-to-nuclear envelope distance (shades of blue). While in cytoplasmic pixels near the NE, 89% of the experiments (N = 25) show two correlation maxima (two dynamic mechanisms, light blue), in pixels far from the NE only 43% of them (N = 13) show two correlation peaks (dark blue). The situation is different for pixels that correlate nucleus to nucleus regions (shades of red) where the percentage of curves that show two correlation peaks is 76% (N = 22) independently on the pixel position relative to the NE. All together these results show that the dominant mode of C protein motion throughout the cytoplasm and nucleus is by free diffusion. However, DENV C protein dynamics exhibits an additional low mobility population of proteins probably due to local interactions with cellular components as previously suggested in literature^13–15^.

### Nucleocytoplasmic DENV C protein transport

In the previous section by spatially cross-correlating pixels (columns) within the same cellular compartment we measured the mobility of C protein in the nucleus and cytoplasm of DENV infected cells. In this section, we address the nucleocytoplasmic transport of C protein by spatially cross-correlating pixels across the NE.

It is well known that, in eukaryotic cells, transport of proteins between the cytoplasm and the nucleus is a major process that occurs through nuclear pore complexes (NPCs). NPCs are the exchanging sites of molecules between the two compartments and are distributed across the NE. The NPC constitutes a gate, of about 8 to 10 nm in diameter, for passive diffusion. Thus, small proteins (10–15 kDa) can freely diffuse through the pores, whereas large molecules and complexes (about 40 kDa) exhibit a delayed or even a prevented translocation mechanism (for proteins > 60 kDa)^16^. Consequently, proteins above the limit size for passive diffusion can enter the nucleus only by an active carrier-mediated transport. To cross the NPC, each cargo must contain defined nuclear localization signals, which are recognized by specific import receptors to transport/recruit specific proteins into the nucleus. Shortly after traversing the NPC the receptors release their cargo and recycle, moving back to the cytoplasm to start a new transport cycle.

There are two main processes linked to DENV C protein function: the release of the viral genome during infection and the recruitment of the viral RNA during assembly^13,14^. Although particle assembly takes place on ER membranes, fluorescence microscopy shows that DENV C localizes both in the cytoplasm (ER and lipid droplets) and in nucleus (nucleoli) of infected cells^15^. Why and how the capsid protein accumulates in these locations are still open questions. It has been previously reported that, like other flavivirus proteins^17,18^, the capsid protein localizes in the nucleoli of cells, independently of viral infection, and it is actively transported into the nucleus^19^. Three nuclear localization signals (NLSs) have been proposed in DENV2 capsid protein, which consist mainly of patches of basic residues that when mutated, nuclear localization is reduced^20^. Studies in DENV and in other viruses such as Japanese encephalitis virus, also suggest that there may be a correlation between nuclear localization and viral replication, indicating there might be an additional function of the capsid protein localization in virus production^20–22^. However, the mechanisms and functional significance of capsid accumulation in the nucleus are still to be determined.

Beside the unknown biological function of capsid subcellular accumulation/localization, the conserved feature of this protein to accumulate in the nucleus across flaviviruses supports a relevance^23,24^. An outstanding question is whether the C protein that enters in the nucleus is also substrate for encapsidation. Thus, it is relevant to address whether it moves only from cytoplasm-to-nucleus or moves in a bidirectional fashion. To examine these possibilities, we applied the pair correlation approach among pixels (columns in the intensity kymogram) crossing the NE in both directions (from cytoplasm-to-nucleus and from nucleus-to-cytoplasm). If DENV C protein crosses the NE in any direction, an arch of delayed diffusion in the pCF kymogram will be expected. The arch is a visual indication of the delay in protein movement due to the presence of a barrier to diffusion (i.e., the presence of the NE). On the contrary, in case DENV C protein is unable to cross the NE (if the NE were impenetrable for C protein diffusion), a lack in the correlation function will be expected^11^. We performed a bidirectional analysis on each measurement to quantify C-protein dynamics considering both directions (from cytoplasm-to-nucleus and from nucleus-to-cytoplasm) and looking at the presence and position of arches to delayed transport.

**Figure 7:**
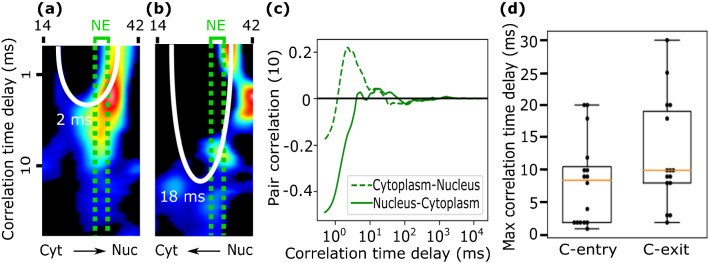
pCF analysis reveals C protein import and export. A pCF analysis of nucleocytoplasmic C-DENV transport shows the C-protein shuttles in both directions. From the complete intensity kymogram we select the region (pixels 14 to 42) in the cytoplasm (or nucleus) that at a distance of 10 pixels entirely correlates with the nucleus (or cytoplasm). (**A**) pCF kymograms analyzed in a direction from cytoplasm to nucleus (dashed green line in **C**). (**B**) pCF kymograms analyzed in a direction from nucleus to cytoplasm (solid green line in **C**). (**C**) Average pCF(10) of lines crossing NE calculated for the kymograms shown in (**A**,**B**). The transport of C-protein from cytoplasm-to-nucleus is favored compared with that of C-protein diffusing in the opposite direction as indicated by the amplitude of the pCF peaks. (**D**) Box plot for each direction, C-entry indicates cytoplasm to nucleus direction with an interquartile range of 2 to 11 ms. C-exit indicates nucleus to cytoplasm direction with an interquartile range of 8 to 19 ms.

Figure 7 shows a representative result of the pCF(10) curve computed in the cytoplasm-to-nucleus, or C-entry, direction (dashed green line) and in the opposite, nucleus-to-cytoplasm, or C-exit, direction (solid green line). In both analyzed directions, pCF kymograms clearly show a lengthening of the time of the correlation, compared to intranuclear and intracytoplasmic diffusion. Interestingly, we observed C transport not only from cytoplasm-to-nucleus (N = 17) but also from the nucleus back to the cytoplasm (N = 17) thus revealing, for the first time, C protein bidirectional translocation dynamics.

We found that the average transport times across NE is in the range of 2 to 11 ms for C-entry (cytoplasm-to-nucleus direction) and 8 to 19 ms for C-exit (nucleus-to-cytoplasm direction). The fact that the measured correlation time in both directions is not significantly different suggests on average similar translocation processes. The timing observed agrees with fast active translocation of DENV C, similar to that observed with other proteins analyzed by single particle tracking experiments^25–28^.

Finally, as observed from the pCFs peaks’ amplitudes in Fig. 7C, the fraction of C protein diffusing from cytoplasm-to-nucleus is ≈ 5 times higher compared to that of a C protein diffusing in the opposite direction, suggesting that C protein import is more efficient as given by the ratios of the peaks’ amplitudes. Interestingly, we found a favored direction (either C-entry or C-exit) in 76% of the measurements (13 from 17) where the ratios of the pCFs’ amplitudes were more than 2 times greater in either direction. Overall, we determined that in 60% of the measurements with a defined favored direction, nuclear C protein import is favored while in the remaining 40% the nuclear C protein export is the favored direction.

Taken together, the pCF analysis applied here, provides unique quantitative information about DENV C protein mobility in subcellular compartments and their transport mechanism across the NE of infected cells.

### Simulations

**Figure 8:**
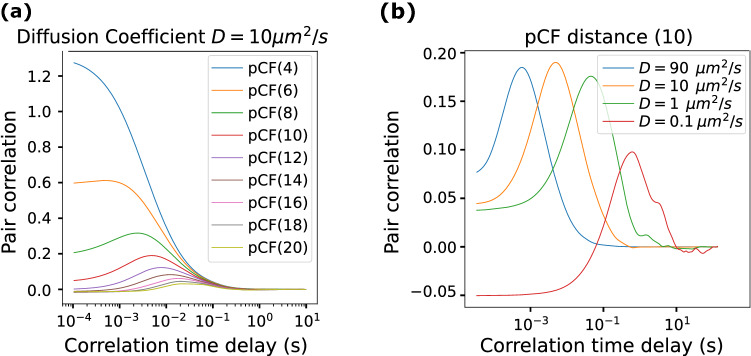
pCF simulated data. To build confidence on the interpretation of our experimental results, we simulated (SimFCS, www.lfd.uci.edu) different scenarios where molecules (proteins) diffuse in a 64 pixels (1 pixel = 50 nm) cubic box with defined diffusion coefficients and molecular brightness. (**A**) pCFs at different distances from 4 to 20 pixels are calculated and displayed in different colors. The maximum of the pCF curve moves at longer correlation times while the amplitude decreases as the computed distance increases. (**B**) pCFs calculated at a distance of 10 pixels for different values of the diffusion coefficient. The maximum of the pCF(10) moves at longer correlation times as the diffusion coefficient decreases.

To build confidence on the interpretation of our experimental results, we simulated (SimFCS, www.lfd.uci.edu) a situation where 200 molecules (proteins) diffuse in a 64 pixels (1 pixel = 50 nm) cubic box with defined diffusion coefficients and molecular brightness 10^6^ counts per second per molecule. The pixel dwell time was set at 1 μs and the line length was set at 32 pixels. It is important to carefully set the line time (pixel dwell time * pixels along the line) to be fast enough so that the same particle (protein) is observed when the same pixel is measured in the next scanned line. During this simulation, for each pCF distance, all the available columns of the 5 million simulated lines were averaged to obtain the curves plotted in Fig. 8. Panel A shows pCFs at different pixel distances from 4 to 20. As expected, the correlation time increases while the correlation amplitude decreases with the distance. Panel B shows the pCF(10) calculated at a distance of 10 pixels for 4 different values of the diffusion coefficient. The maximum of the pCF(10) moves at longer correlation time delays as the diffusion coefficient decreases.

**Figure 9:**
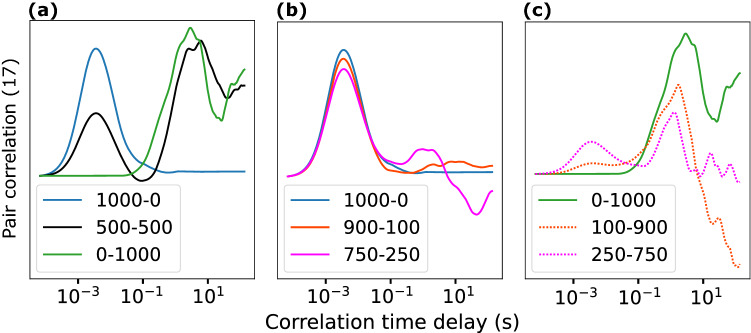
pCF simulated data for two coexisting molecular species. Two coexisting molecular species diffuse at 50 μm^2^/s (fast population) and 0.1 μm^2^/s (slow population) in a cubic box of 64-pixels size. Labels indicate the number of molecules in a format: fast molecules-slow molecules. The total number of molecules was kept constant across the simulations at 1000 molecules/box. (**A**) The pCF analysis shows two peaks (black curve) with correlation times delays at 3.7 ms and 950 ms corresponding to the average times required by each molecular population to travel between the two positions (17 pixels or 850 nm apart). When only one molecular population is present (blue and green curves), a single peak is detected. (**B**,**C**) Effect of relative populations on the amplitude of the correlation curve. If either of the two populations is less than ≈25%, the pCF analysis could not clearly separate them and the recovered correlation function tends to emphasize the largest population.

We also simulated a situation where two coexisting molecular species (proteins with different mobility properties) diffuse in a 64 pixels cubic box with defined diffusion coefficients with an identical brightness (10^7^ counts per second per molecule). The diffusion coefficients were chosen on the order of those obtained along the experiments (tens of μm^2^/s for the fast component and a fraction of μm^2^/s for the slow component). The pixel dwell time was set at 1 μs and the line length was set at 64 pixels. Across the performed simulations, we changed the relative concentration of each of the two protein populations while the total number of molecules was maintained constant (1000 molecules/box), Fig. 9.

For each simulated condition, 3 million simulated lines were averaged to obtain the plotted pCF (17) curves. Figure 9A shows that two pCF peaks appear when two populations of molecules are present (black curve), and the correlation distance, δr, is above the resolution of the microscope (approx. 6 pixels, corresponding to 300 nm, in our microscope and simulations) in total agreement with our experimental results. On the contrary, if only one molecular species is present (blue and green curves) a single peak in the pCF with correlation times 3.7 ms for the fast, and 950 ms for the slow mobility populations is observed.

Finally, changing the relative number of particles of each species modifies the amplitude of each of the obtained pCF peaks thus showing the effect of relative populations on the amplitude of the correlation curve, Fig. 8B,C. If either of the two populations is less than ≈25%, it is difficult for the pCF analysis to separate them and thus the recovered correlation function tends to emphasize the largest population.

## Conclusions

In this work, we reveal molecular dynamics and protein translocation by applying the pair correlation function method (pCF) to the diffusion of dengue virus C-protein in infected cells. To do so, a DENV C-mCherry fusion was constructed and then transfected in BHK-21 live cells. We demonstrated that DENV C-protein moves between the nucleus and cytoplasm in both directions, which is, as far as we know, the first experimental evidence on bidirectional nucleocytoplasmic shuttling of DENV C protein. By cross-correlating pixels at different compartments, we found that the time taken to cross the NE differs depending on transport direction. Additionally, by cross-correlating pairs of pixels at the same subcellular compartment, we have measured DENV C protein dynamics both in the cytoplasm and in the nucleus of infected cells. Our findings indicate that, in both compartments, DENV C protein shows a two-population behavior: one population of slow mobility and one population of fast mobility. When the intra-compartment fast mobility mechanism (the short median time required for proteins to travel between the two positions) are translated into diffusion coefficients, both cytoplasmic (3 ms, Dapp = 38 μm^2^/s) and nuclear proteins populations (5 ms, Dapp = 23 μm^2^/s) agree with diffusion coefficients previously reported from correlation analysis. Our results show that the dominant mode of C protein motion throughout the cytoplasm and nucleus is by free diffusion. However, DENV C protein dynamics exhibits an additional low mobility population of proteins probably due to local interactions with cellular components.

By using the sensitivity of pCF approach on data analysis direction, we were able to determine DENV C protein transport during nuclear entry (cytoplasm-to-nucleus direction) and nuclear exit (nucleus-to-cytoplasm direction). Our findings show that the average characteristic time for transporting C protein across NE is between few and tens of milliseconds for both the cytoplasm-to-nucleus and the nucleus-to-cytoplasm directions respectively. Despite the known structural difference on nuclear localization signals for import and export events, translocation-times exhibit comparable values in both directions suggesting that the molecular dynamics underlying C-shuttling could be similar during nuclear entry and exit. However, further experiments should be made to determine the exact transport mechanism. Interestingly, by analyzing the amplitude of the pCFs curves we found that in 76% of the translocation measurements a favored direction, either C-import or C-export, is observed. From those, in the 60% of the measurements the fraction of C-protein diffusing from cytoplasm-to-nucleus is higher with respect to C-protein diffusing in the opposite direction while in the remaining 40% the situation is the opposite (the nuclear DENV C protein export is the favored direction). Although informative, further experiments need to be performed to understand whether this bidirectional similar behavior of C protein export and import has consequences on viral replication.

In conclusion, pCF analysis of confocal line-scans allowed us to observe the major dynamic features of DENV C protein dynamic at subcellular compartments as well as unveil its bidirectional transport across the NE at single-molecule level and in intact infected-cells. We showed that transport can take place with similar correlation times (at the ms time scale) both during DENV C protein export and import, advancing our current understanding of DENV C dynamics. We believe these results can stimulate further studies on viral C protein nucleocytoplasmic movement using new correlation-based techniques and related methods.

## Materials and methods

### Materials

All inorganic and organic materials were purchased from Sigma-Aldrich.

### Molecular and cell biology methods

#### Construction of recombinant DENV C-mCherry

For constructing the recombinant full-length DENV containing the C-mCherry fusion, we modified a DENV reporter construct that we have previously described that includes a luciferase coding region. We replaced the SacII-SphI fragment of the reporter with a fragment containing mCherry coding sequence fused to the foot and mouth disease virus 2A (FMDV2A) protein, in such a way to obtain the capsid fused to mCherry that would be released from the rest of the viral polyprotein by the FMDV2A cleavage. The fragment used to replace the SacII-SphI region was obtained by overlapping PCR. The new construct named DENV C-mCherry was sequenced and directly used for in vitro RNA transcription. The viral RNA DENV C-mCherry was competent for viral translation and genome replication.

#### Cell culture and virus transfection

Baby hamster kidney cell line (BHK-21, ATCC, CCL-10) was cultured in minimum essential medium alpha (α-MEM) supplemented with 10% fetal bovine serum (Gibco), 100 U/ml penicillin–streptomycin. Plasmids containing DENV WT and DENV C-mCherry full genomes were linearized with XbaI restriction enzymes. Viral capped RNA was in vitro transcribed by T7 RNA polymerase in the presence of m7GpppA. RNA transfections were performed using Lipofectamine 2000 (Thermo Fisher Scientific) and Opti-MEM medium (Gibco) according to the manufacturer’s instructions. 50 ng of viral RNA WT or C-mCherry were transfected into BHK-21 cells grown in 8-well chambered cover-glass plates (Thermo Fisher Scientific). The observations were performed immediately after adding transfection reagent. For the mCherry control experiments, 500 ng of pmCherry-C1 plasmid (Clontech) were transfected into BHK cells using the protocol described above.

### Confocal imaging and data analysis

#### Confocal microscopy of transfected BHK cells

Confocal microscopy measurements were performed by means of a LSM 880 (Zeiss) inverted microscope using a C-Apochromat 40X/1.2 water-immersion objective (Zeiss). All experiments were carried out using a pinhole size of 1 Airy disk unit. During the experiments, cells were kept at 37 °C in a 5% CO_2_ temperature-controlled chamber (Pecon). Excitation wavelength was set at 543 nm using a He–Ne green laser (Lasos) and power intensities chosen to achieve high photon count rate per molecule to maximize the signal/noise ratio but low enough to prevent molecular photobleaching and phototoxicity processes. The measured photon count rates per molecule ranged from 30 to 100 kcps approximately. Fluorescence emission was collected in the range 550–650 nm using a spectral filter (Quasar module) and detection performed by GaAsP detectors (Hamamatsu) in photon counting mode. For line correlation analysis measurements, a line-scanning of the laser beam along 64-pixels (corresponding to distances of 2.6 to 5.3 μm) with a fixed pixel size of 83 nm was used. Typically, a total of 200,000 consecutive lines at 0.47 ms/line were collected for each measurement. Data was analyzed by using the SimFCS software (available at www.lfd.uci.edu) and custom-made code written in Matlab (MathWorks) and Python (Phyton Software Foundation).

### The pair correlation function (pCF) approach

As originally conceived by Digman and coworkers^6^ and more recently extended to the 2D case^7,8,29^, the pCF corresponds to the spatiotemporal cross-correlation spectroscopy occurring between two spatially separated pixels (columns) of the intensity kymogram and can reveal the transport route at the single molecule level at any subcellular compartment and across cellular boundaries^9,10,29–31^. The general mathematical framework for the cross-correlation analysis has been already presented^6^ and is similar to the approach presented by Ries and coworkers^32,33^. The basic idea behind the pCF method is to follow the same molecule moving along the sample by calculating the temporal cross-correlation between a distant pair of points. In the spatial pair cross-correlation approach, the spatial pair cross-correlation functions (pCFs) return the time it takes a molecule to move (diffusing or not) between the two locations, by correlating the fluorescence intensity fluctuations at a pair of specific points (columns) of the fluorescence intensity kymogram. Briefly, the pCF is calculated correlating the intensities along pair of pixels positioned at points $r$ and $r + \delta r$, as:

$$ pCF\left( {\delta r, \tau } \right) = \frac{{\langle{F\left( {r, t} \right)F\left( {r + \delta r, t + \tau } \right)}\rangle}}{{\langle{F\left( {r, t} \right)F\left( {r + \delta r, t} \right)}\rangle}} - 1 $$

where $\tau$ is the time delay between acquisitions of the fluorescence intensity, $F$, at two points (columns) in the scanned line, and the temporal average is indicated by the brackets 〈〉. Finally, either of these three scenarios are possible: A positive pCF peak indicates a correlation of the signals thus demonstrating that some of the molecules encountered in pixel $r + \delta r$ took a time $\tau$ to move a distance $= \delta r$ showing unimpeded dynamic trafficking between those two positions, whereas a negative pCF minimum denote an anticorrelation of the signals and a zero pCF indicates that the molecules encountered between those two positions are different and thus the signals between studied points are independent.

The free unrestrained molecular mobility may seem improbable in the crowded cell environment. Thus, the pCF can be used as a general framework to estimate the apparent mobility and furthermore, to detect time delays with respect to the free diffusion case, highlighting the presence of barriers to molecular diffusion.

In this work, the pCF was calculated from line-scanning measurements perpendicularly crossing the NE interface. Fluorescence intensity measurements and the pair correlation functions were reconstructed in the form of a kymogram in which the x-coordinate represents the position along the line, the y-coordinate corresponds to the correlation time delay, and the amplitude (fluorescence intensity or pCF function amplitudes) are color-coded by a black-to-red color scale. In both cases warmer colors indicate higher amplitudes.

## Supplementary Information

Supplementary Video 1.

Supplementary Information.
