# Author Correction: In vivo pair correlation microscopy reveals dengue virus capsid protein nucleocytoplasmic bidirectional movement in mammalian infected cells

**DOI:** 10.1038/s41598-022-15832-0

**Published:** 2022-07-05

**Authors:** Ignacio Sallaberry, Alexis Luszczak, Natalia Philipp, Guadalupe S. Costa Navarro, Manuela V. Gabriel, Enrico Gratton, Andrea V. Gamarnik, Laura C. Estrada

**Affiliations:** 1 Departamento de Física, Facultad de Ciencias Exactas y Naturales, Universidad de Buenos Aires and IFIBA-National Research Council for Science and Technology (CONICET), 1428 Buenos Aires, Argentina; 2 Fundación Instituto Leloir-National Research Council for Science and Technology (CONICET), 1405 Buenos Aires, Argentina; 3 Laboratory for Fluorescence Dynamics and Beckman Laser Institute and Medical Clinic, University of California, Irvine, CA USA

Correction to: *Scientific Reports* 10.1038/s41598-021-03854-z, published online 24 December 2021

The original version of this Article contained an error in the order of the Figures. Figure 6 was published as Figure 7 and Figure 7 was published as Figure 6. The Figure legends were correct.

The original Figures 6 and 7 and accompanying legends appear below.

**Figure 6:**
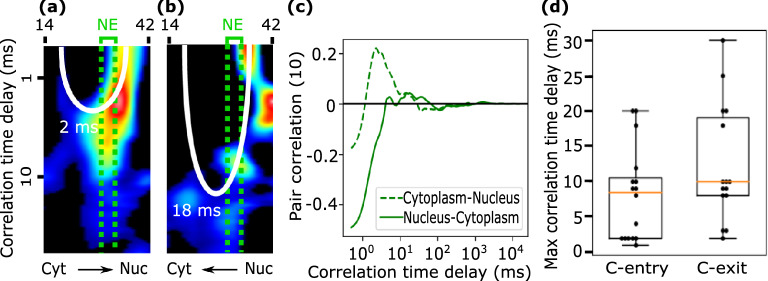
pCF pixel-by-pixel analysis Representative results of a pCF(10) analysis calculated pixel-by-pixel along a scanned confocal line across the cytoplasm (left) and the nucleus (right) of a DENV infected cell. Panels (**A**,**B**) show pCF(10) curves as a function of the NE distance (the closer to the NE, the lighter the color). Pixels closer to each other (similar shades of blue or red) tend to have similar correlation curves. In panels (**C**,**D**) pCF(10) curves at 1 and 2 μm from NE are highlighted showing the presence of positive correlation peaks close to the NE (in light color) but the presence of only one positive correlation peak far from the NE (in dark color).

**Figure 7:**
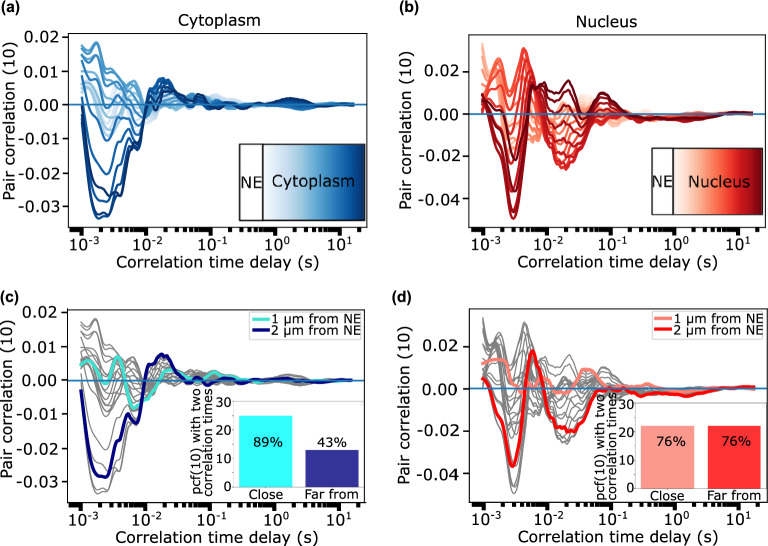
pCF analysis reveals C protein import and export. A pCF analysis of nucleocytoplasmic C-DENV transport shows the C-protein shuttles in both directions. From the complete intensity kymogram we select the region (pixels 14 to 42) in the cytoplasm (or nucleus) that at a distance of 10 pixels entirely correlates with the nucleus (or cytoplasm). (**A**) pCF kymograms analyzed in a direction from cytoplasm to nucleus (dashed green line in **C**). (**B**) pCF kymograms analyzed in a direction from nucleus to cytoplasm (solid green line in **C**). (**C**) Average pCF(10) of lines crossing NE calculated for the kymograms shown in (**A**,**B**). The transport of C-protein from cytoplasm-to-nucleus is favored compared with that of C-protein diffusing in the opposite direction as indicated by the amplitude of the pCF peaks. (**D**) Box plot for each direction, C-entry indicates cytoplasm to nucleus direction with an interquartile range of 2 to 11 ms. C-exit indicates nucleus to cytoplasm direction with an interquartile range of 8 to 19 ms.

The original Article has been corrected.

