# Supplementary material for: In vivo pair correlation microscopy reveals dengue virus capsid protein nucleocytoplasmic bidirectional movement in mammalian infected cells
Source: Sci Rep. 2021 Dec 24;11:24415. doi: 10.1038/s41598-021-03854-z (PMC8709865; doi:10.1038/s41598-021-03854-z)
Supplement: Supplementary file 2 — Supplementary Information. [file 41598_2021_3854_MOESM2_ESM.docx]

**Supplementary information**

Immunofluorescence analysis using specific antibodies against C protein for the WT virus reproduced that observed with the C-mCherry confocal images showing C-mCherry accumulation in the cytoplasm, nucleolus, and lipid droplets of the infected cells. We conclude that C-mCherry is suitable to investigate C protein nucleocytoplasmic shuttling and intracompartment dynamics during a viral replication cycle.
